# Validation and cultural adaptation of the pregnancy-adapted Mediterranean Diet Adherence Screener (ITpreg-MEDAS) in Italian pregnant women

**DOI:** 10.1007/s00394-026-04087-4

**Published:** 2026-08-29

**Authors:** Silvia Callegaro, Francesca Scazzina, Pedro Mena, Christine Tita Kaihura, Andrea Dall’Asta, Ramon Estruch, Rosa Casas, Alice Rosi

**Affiliations:** 1 Human Nutrition Unit, Department of Food and Drug, University of Parma, Parma, Italy; 2 Dipartimento Cure Primarie, U.O.C. Salute Donna, Azienda Unità Sanitaria Locale di Parma, Parma, Italy; 3 Department of Medicine and Surgery, Obstetrics and Gynecology Unit, University of Parma, Parma, Italy; 4 Centro de Investigación Biomédica en Red de Fisiopatología de la Obesidad y Nutrición (CIBEROBN), Madrid, Spain; 5 Institut de Recerca en Nutrició i Seguretat Alimentaria (INSA- UB), University of Barcelona, Barcelona, Spain; 6 Department of Internal Medicine Hospital Clinic, University of Barcelona, Barcelona, Spain; 7 Institut de Recerca August Pi Sunyer (IDIBAPS), Barcelona, Spain

**Keywords:** Pregnancy, Mediterranean diet, Index, Translation, Cross-cultural adaptation, Validated questionnaire

## Abstract

**Purpose:**

The beneficial effects of Mediterranean Diet (MD) during pregnancy are well established. However, validated tools adapted to assess adherence to the MD during pregnancy and across different cultural contexts remain limited. This study aimed to translate, cross-culturally adapt, and validate an Italian version of the pregnancy-adapted Mediterranean Diet Adherence Screener (ITpreg-MEDAS), derived from the original Spanish preg-MEDAS questionnaire. We hypothesized that the ITpreg-MEDAS would show adequate construct validity and would be associated with markers of diet quality during pregnancy.

**Methods:**

The preg-MEDAS is a 17-item questionnaire designed to assess adherence to the MD among pregnant women. The Italian version was developed following a six steps process in accordance with established methodological guidelines: forward translation, synthesis I, blind-back translation, synthesis II, pre-testing, and validation. Psychometric properties were evaluated by assessing internal consistency, test-retest reliability, and construct validity, in a sample of Italian pregnant women.

**Results:**

The preg-MEDAS was translated and cross-cultural adapted, requiring some semantic revisions. All 25 Italian pregnant women involved in the pre-testing in pre-testing found it clear and comprehensible. A total of 116 Italian pregnant women participated in the validation phase, with a mean ITpreg-MEDAS score of 7.2 ± 2.5 out of 17, indicating medium adherence to the MD. Reliability was confirmed by good test-retest correlation (*r* = 0.756) and acceptable internal consistency (Cronbach’s α = 0.623).

**Conclusion:**

The ITpreg-MEDAS is a reliable and valid Italian version of the preg-MEDAS questionnaire, providing a simple tool to assess adherence to the Mediterranean Diet during pregnancy in both clinical practice and research.

**Supplementary Information:**

The online version contains supplementary material available at https://doi.org/10.1007/s00394-026-04087-4.

## Introduction

Adequate maternal nutrition during pregnancy is essential for the health and well-being of both the mother and the offspring [1, 2]. Proper eating habits and a healthy nutritional status during pregnancy are associated with a lower risk of adverse short- and long-term health outcomes for the mother-child dyad, including gestational diabetes mellitus, hypertensive disorders, excessive gestational weight gain, preterm birth and impaired foetal growth, with resulting reduced risk of developing non-communicable chronic diseases later in adult life [3–5]. Given these premises, it is important that, during pregnancy, the future mother follows a dietary pattern that has beneficial effects on the health of both mother and foetus.

The Mediterranean Diet (MD) is a well-known dietary pattern typical of the inhabitants of the Mediterranean basin, characterised by the use of olive oil as source of fats, high consumption of vegetables, fruits, nuts, legumes, and whole grain cereals, moderate consumption of fish and dairy products, poultry and eggs, and a low consumption of red and processed meats and sweets [6, 7]. The role of MD in preventing non-communicable diseases and reducing mortality is well-established [8–11]. More recently, a growing body of evidence, including a randomized controlled trial, has also highlighted the benefits of adhering to the MD during pregnancy for both maternal and offspring health. Several studies have reported an association between the Mediterranean dietary pattern and a reduced risk of gestational diabetes mellitus [5, 12–16], gestational weight gain [13, 17, 18], pregnancy-induced hypertension, and pre-eclampsia [5, 14, 15]. In addition, adherence to the MD has been associated with improved neonatal outcomes, including a lower risk of fetal growth restriction in utero [5, 15, 19, 20] (15,20) and low birth weight [5, 15] as well as preterm birth [5, 14, 15, 20]. Moreover, the MD plays a protective role against the development of congenital and cardiometabolic defects in the foetus, as well as allergic disorders, such as atopy, asthma, and eczema later in childhood [5]. Beyond metabolic and perinatal outcomes, emerging evidence suggests that adherence to the MD during pregnancy may positively influence maternal mental health and well-being. Indeed, a number of studies have reported an association between the MD and improved sleep quality, along with a reduction in maternal anxiety and stress [12, 21] and postpartum depression [22].

Despite the well-established benefits of the MD, validated tools specifically adapted for assessing adherence during pregnancy and across different cultural contexts remain limited. Pregnancy entails specific nutritional recommendations [23] that may not be fully captured by standard MD scores [24]. We hypothesized that a pregnancy-adapted Mediterranean Diet Adherence Screener [25], culturally adapted to the Italian context, would provide a valid measure of diet quality during pregnancy. Therefore, the aim of this study was to culturally adapt and validate the Italian version of the pregnancy-adapted Mediterranean Diet Adherence Screener (ITpreg-MEDAS) in pregnant women.

## Methods

### Pregnancy-adapted Mediterranean Diet Adherence Screener (preg-MEDAS)

The preg-MEDAS [25] questionnaire comprises 17 items that assess the frequency of consumption of various food groups, including extra virgin olive oil, vegetables, fruits, *sofrito*, whole-grain cereals, refined cereals, pulses, fish and seafood, fatty fish, red meat, processed meat, white meat, carbonated and/or sugar-sweetened beverages, nuts, sweets, milk and dairy products, and animal fats such as butter, margarine, and cream. For each item, daily or weekly consumption thresholds were defined according to the principles of the MD, adapted to specific nutritional requirements of pregnancy. Responses consistent with these recommendations are assigned 1 point, whereas responses that do not adhere to the MD recommendations receive 0 points. In particular, 1 point is attributed to the consumption of ≥ 4 tablespoons/day of extra virgin olive oil; ≥3 servings/day of vegetables; ≥2 fruits/day; ≥2 servings/week of *sofrito*, ≥ 5 servings/week of whole-grain cereals; ≥3 servings/week of pulses; ≥3 servings/week of fish and seafood; ≥1 serving/week of fatty fish; ≥3 servings/week of chicken, turkey, rabbit, or lean pork; ≥3 servings/week of nuts; and ≥ 3 servings/day of milk and dairy products. Conversely, 1 point was assigned when the intake of refined cereals was < 3 servings/week; ≤1 serving/week of red meat; ≤1 serving/week of processed meat; <1 serving/week of sugar-sweetened or carbonated beverages; <2 servings/week of sweets; and < 1 serving/week of animal fats (such as butter, margarine, or cream). The total adherence score is calculated by summing the score obtained in all items and ranges from 0 to 17 points. Based on the total score, respondents are classified into three adherence levels: low (≤ 6 points), medium (7–11 points), and high (≥ 12 points) adherence to the MD.

### Translation and cross-cultural adaptation

**Fig. 1:**
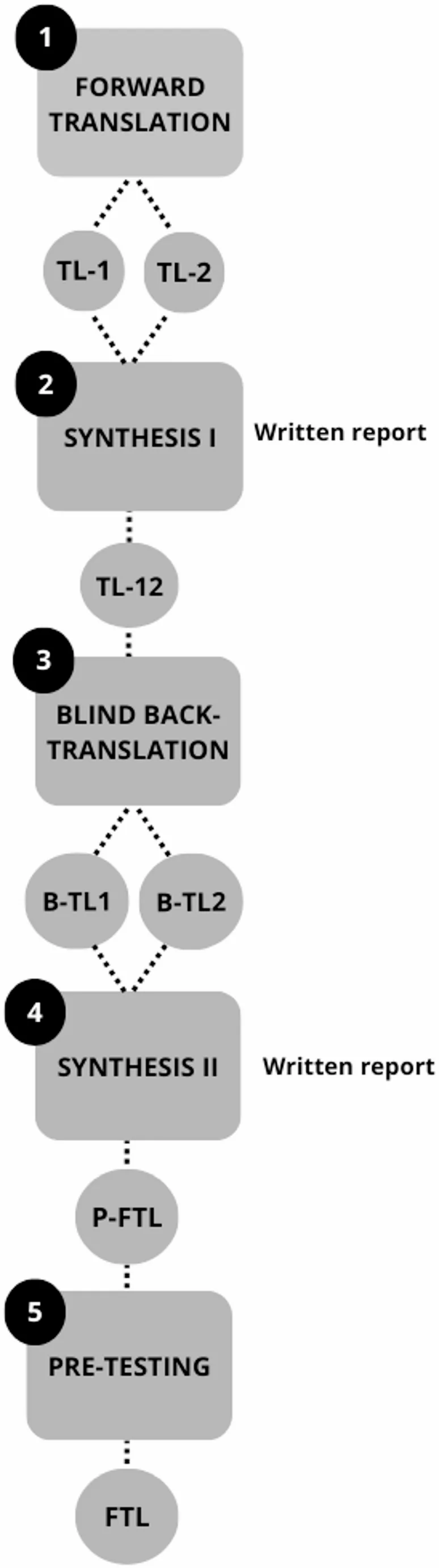
Phases of the translation and cross-cultural adaptation process for the development of the Italian version of the preg-MEDAS questionnaire

The translation and cross-cultural adaptation process was conducted in accordance with the guidelines proposed by Beaton et al. [26] and Sousa et al. [27], including the following steps: (1) *forward translation*, (2) *synthesis I*, (3) *blind back-translation*, (4) *synthesis II*, and (5) *test of the prefinal version of the instrument* as reported in Fig. 1.

The first step (*forward translation*) entailed the translation of the instrument from the original language (Spanish) into the target language (Italian). This phase was carried out by two independent translators, both native Italian speakers, bilingual (fluent in both Italian and Spanish) and bicultural (with in-depth experience of the Spanish and Italian cultures), but with different professional backgrounds. One translator had expertise in nutrition and health-related terminology and was familiar with the construct measured by the instrument, while the other translator was knowledgeable about Italian linguistic and cultural nuances but had no prior knowledge of nutrition terminology or of the construct. At the end of this phase, two forward-translated versions of the instrument (TL-1 and TL-2) were produced. In the second step (*synthesis I*), the two translators and a recording observer convened to synthesise the results of the translations, working on the original questionnaire and on the two translated versions. This process resulted in a preliminary Italian version of the instrument (TL-12), and in a written report that documented the synthesis process and the resolution of the discrepancies between the two translations. The third step involved the production of two back-translated versions of the instrument from Italian to Spanish. This phase involved two independent translators whose native language was Spanish. Both translators were bilingual and bicultural, and had different professional backgrounds, as described in the first phase. Importantly, both translators were blinded to the original version of the instrument. At the end of this phase, two back-translated versions in Spanish were produced (B-TL1 and B-TL2). In the fourth step (*synthesis II*) an expert multi-disciplinary committee, comprising the four translators, one member of the research team, and one health care professional who is familiar with the content area, reviewed the two back-translations. The committee evaluated the instructions, items, and response format with respect to wording, grammatical structure, semantic equivalence, conceptual meaning, and content relevance, with the aim of establishing conceptual, semantic, and content equivalence of the instrument. Any discrepancy identified between the two back-translations (B-TL1 and B-TL2), as well as between each back-translation and the original Spanish version, was discussed and resolved by consensus. This phase resulted in the development of the pre-final Italian version of the instrument (P-FTL), together with a written report documenting the identified issues and the rationale underlying the decisions made.

The fifth phase involved the administration of the pre-final Italian version of the instrument in the target population to assess the clarity and comprehensibility of the tool. A brief questionnaire with a dichotomous scale (clear/unclear) was also administered. In particular, the survey was composed of twenty questions investigating the clarity of the response format (how the response is delivered), individual items, and the questionnaire in general. In addition, participants were invited to provide suggestions for improving the overall clarity and comprehensibility of the tool. According to guidelines [27], the sample size requested was between 10 and 40 subjects. Any instruction, response format, or item deemed unclear by at least 20% of the sample was subjected to re-evaluation. Last, the final version (FTL) of the Italian pregnancy-adapted Mediterranean Diet Adherence Screener (ITpreg-MEDAS), together with the complete documentation of the translation and cultural adaptation process, was submitted to the original authors for a final revision and approval of the methodological procedures.

### Validation of the ITpreg-MEDAS

The psychometric properties of the translated and cross-culturally adapted instrument were validated in a sample of Italian pregnant women. Participants were recruited, from April 2025, through the distribution of flyers, in key areas of interest for pregnant women, including antenatal classes, counselling centres, and public and private gynaecology and obstetrics clinics. In addition, the study was promoted through major communication and social media platforms. Inclusion criteria were being pregnant at the time of questionnaire administration, being over the age of eighteen, and being of Italian nationality. The questionnaire was administered via EUSurvey, a dedicated European platform for the creation and dissemination of online surveys and forms [28]. Data were collected anonymously, ensuring that no personal data, including the IP address, were stored by the platform. Furthermore, to guarantee anonymity, participants were identified using alphanumeric codes, and all data were analysed in aggregated form. Informed consent was obtained at the beginning of the questionnaire and participants were allowed to proceed with the completion of the ITpreg-MEDAS only after selecting the “I agree” option.

For validation purposes, the questionnaire was administered to participants on two separate occasions under identical conditions, with a two-week interval between administrations. This timeframe is considered sufficiently long to minimise recall of specific responses while being sufficiently brief to prevent changes in dietary habits [29]. Based on previous literature, questionnaire validation studies require a sample size corresponding to a minimum of six to ten participants per item [29]. Given that the ITpreg-MEDAS questionnaire consisted of 17 items, the estimated sample size required for its validation ranged from 102 to 170 participants. During the first administration, the questionnaire began with a set of screening questions to ascertain compliance with the inclusion criteria. This was followed by a section collecting sociodemographic information for a complete classification of the participants. This section included questions on: gestational age at the time the questionnaire was administered, maternal age, geographical area of birth in Italy (Northwest, Northeast, Central, South, or Islands), educational level (pre-graduated, graduated, or post-graduated), nutritional background (having attended a university course on topics related to food, dietetics, and/or nutrition), employment status (unemployed, part-time, full-time, student, or maternity leave), and parity. Participants were also requested to provide an email address to which the link to the second questionnaire administration could be sent.

### Statistical analysis

The primary outcome of the study was the assessment of the validity of the ITpreg-MEDAS. Secondary analyses were considered exploratory in nature. No formal adjustment for multiple comparisons was applied due to the exploratory nature of secondary analyses.

A descriptive analysis was conducted to describe the socio-demographic profile of the participants. Continuous variables were expressed using mean and standard deviation, whereas categorical variables were reported as absolute values and percentages. The internal consistency of the ITpreg-MEDAS was assessed using Cronbach’s alpha, and a coefficient value above 0.60 was considered acceptable [29, 30]. The temporal stability (test-retest reliability) was investigated through Pearson’s correlation coefficient and the intraclass correlation coefficient (ICC) between total scores obtained at baseline (t1) and at follow-up (t2). Furthermore, the construct validity was evaluated by applying Welch’s independent-samples t-test to compare ITpreg-MEDAS scores between participants with and without a background in nutrition, using data from the first administration of the questionnaire (t1). All statistical analyses were performed using the Statistical Package for Social Science (IBM SPSS Statistics, version 29.0.1.0, IBM Corp., Armonk, NY, USA) and statistical significance was set at *p* < 0.05.

### Bias and confounding control

Potential confounding factors were addressed through ANCOVA model to compare the total ITpreg-MEDAS scores between participants with and without a nutritional/health background, while controlling for the effect of relevant sociodemographic variables (educational level, parity, area of birth, occupation) as covariate. Data collection followed standardized procedures to minimize information bias, and quality control checks were applied to reduce the risk of data entry errors.

## Results

### Translation and cross-cultural adaptation

The forward translations (TL-1 and TL-2), the synthesised version (TL-12), the back-translations (B-TL1 and B-TL2), and the pre-final Italian version of the preg-MEDAS questionnaire (P-FTL) are presented in Table S1 (Supplementary material). In the *forward translation* phase, where two independent translators produced two Italian translations (TL-1 and TL-2) of the preg-MEDAS questionnaire, the translations were found to be similar for the majority of items. The few discrepancies identified did not concern the semantic meaning of the items but rather pertained to semantic choices.

During the second phase, a researcher involved in the project, together with the two translators, reviewed and discussed the proposed translations and produced a synthesised version (TL-12), resolving all the discrepancies emerging from TL-1 and TL-2. In particular, the discussion focused on the translation of the Spanish term *“raciones”* (servings), which in Italian can be translated as either *“porzione”* or *“razione”*. The panel agreed to retain the term *“porzione”*, as it is more consistent with common usage and with adherence questionnaires already available in the scientific literature. Another discrepancy concerned the translation of the Spanish expression *“fuego lento”* (simmer) in item 4, which could be translated as either *“a fuoco lento”* or *“a fuoco basso”*. The expression “*a fuoco basso*” was selected, as it is more commonly used in everyday Italian language. During synthesis I, the clarification *“molluschi e crostacei”* (molluscs and crustaceans) was added to item 8 to ensure that both categories were considered when completing the questionnaire, thereby preventing the inclusion of only one of the two. In item 9, the term *“pesce azzurro di piccola taglia”* was retained, as it is consistent with the terminology adopted in the Italian Food-Based Dietary Guidelines (FBDGs) [31]. Additionally, examples of fish belonging to this category were included to facilitate comprehension for respondents. In item 10, *“buey”* (ox) was removed from the examples of red meat, as it is not widely consumed in the Italian population. For item 11, a discussion arose regarding whether to translate the Spanish term *“embutidos”* (cured meats) as *“salumi”* or *“insaccati”*. The term *“salumi”* was preferred, as in Italian it encompasses both products encased in natural or artificial casings (e.g., salami, cotechino, sausages) and processed meats without casings, such as cured ham. In item 12, the specification *“di maiale”* (pork) was added, since the Spanish examples of lean meat (*“lomo”* and *“solomillo”*) refer to lean cuts of pork. Moreover, in the description of the reference portion, *“carne de pollo”* (poultry meat) was translated as *“carne bianca”* (white meat) to include all meat categories mentioned in the item. In item 13, the Spanish term *“refrescos”* (soft drinks) was translated as *“bibite”*, while in item 14 the expression *“frutta secca a guscio”* (nuts) was retained for *“frutos secos”* to avoid confusion with dried fruit. In item 15, *“repostería”* was translated as *“dolci”* (desserts), and *“dulces”* as *“pasticcini”* (pastries).

In the third phase, the synthesised version (T-12) was submitted to two independent back-translators who produced two back-translated versions (B-TL1 and B-TL2). These versions demonstrated a high degree of consistency with the original questionnaire and required only minor lexical adjustments during the fourth phase. The expert multi-disciplinary committee, consisting of the four translators and two additional experts involved in the study, participated in this phase and consolidated the pre-final version of the questionnaire (P-FTL). Specifically, in item 4, the term *“verdure”* (vegetables) was replaced with *“vegetali”*, as it was considered more coherent with Italian culinary terminology. In item 11, following the recommendation of the Spanish native-speaking translators, the term *“würstel”* (sausage) was added among the examples of processed meat to better reflect the meaning of the Spanish term *“salchichas”*. The term *“salsiccia”* was nevertheless retained, as it represents a traditional Italian processed meat product. Finally, based on the comparison of the back-translations, in item 15 the term *“budino”* (pudding) was replaced with the more general expression *“dolci al cucchiaio”* (spoon desserts).

A total of 25 Italian pregnant women, with an age between 18 and 40 years, participated in the evaluation of the clarity and comprehensibility of the P-FTL of the ITpreg-MEDAS questionnaire. The response format was judged to be clear by 96% of participants, with only one subject reporting difficulty in estimating the weekly or daily frequency of consumption of foods and food groups included in the questionnaire. Regarding the individual items, 11 out of 17 were rated as comprehensible by 100% of the participants. The remaining six items, specifically items 1, 2, 6, 9, 11 and 12, were considered clear by 96% of respondents, with only one participant per item reporting difficulties in comprehension. The main issue reported concerned the difficulty in comparing the habitual food intake with the reference portion sizes provided in the questionnaire. However, no specific suggestions were proposed to improve the clarity of individual items. Overall, the questionnaire was found to be clear by 100% of participants. Three participants provided suggestions to improve the overall clarity of the questionnaire. In particular, it was proposed to include, for each item, pictures of the foods and corresponding portion sizes to facilitate questionnaire completion. Additional suggestions included expanding the range of food examples provided for each item and adding a comment box for each question to allow respondents to explain their answers, particularly in cases where entire food groups (e.g., fish) had been avoided during pregnancy due to pregnancy-related symptoms such as nausea. Given that more than 80% of participants evaluated positively the questionnaire and its items as comprehensible, no further revisions of the instrument were considered necessary. The final version of the ITpreg-MEDAS is reported in Table 1.

**Table 1:** Final version of the Italian pregnancy-adapted Mediterranean Diet Adherence Screener (ITpreg-MEDAS)

Punteggio di Aderenza alla Dieta Mediterranea:	Risposta
**1.** Quanti cucchiai di olio d’oliva consuma in totale al giorno? (includendo quello usato per cucinare, friggere, i pasti consumati fuori casa, le insalate, etc.)	≥ 4 cucchiai = 1 punto
**2.** Quante porzioni di verdura e ortaggi consuma al giorno? 1 porzione = 200 g (contorni = ½ porzione)	≥ 3 = 1 punto
**3.** Quanti frutti consuma al giorno?	≥ 2 al giorno = 1 punto
**4.** Quante volte a settimana consuma vegetali cotti, pasta, riso o altri piatti conditi con salsa di pomodoro, aglio, cipolla o porro cotta a fuoco basso con olio di oliva (soffritto)?	≥ 2 alla settimana = 1 punto
**5.** Quante porzioni di cereali e alimenti integrali (pane, riso, pasta) consuma a settimana?	≥ 5 alla settimana = 1 punto
**6.** Quante porzioni di pane, riso e/o pasta raffinati (non integrali) consuma a settimana?	< 3 alla settimana = 1 punto
**7.** Quante porzioni di legumi consuma a settimana? (1 piatto o porzione = 150 g)	≥ 3 alla settimana = 1 punto
**8.** Quante porzioni di pesce o frutti di mare (molluschi e crostacei) consuma a settimana? (1 piatto, pezzo o porzione = 100–150 g di pesce; 4–5 pezzi o 200 g di frutti di mare)	≥ 3 alla settimana = 1 punto
**9.** Consuma una porzione (100–150 g) di pesce azzurro di piccola taglia (es. sardine, sgombro) a settimana?	Si = 1 punto
**10.** Quante porzioni di carne rossa (vitello, manzo, cavallo, puledro, agnello, parti non magre del maiale o anatra) consuma a settimana (porzione = 100–150 g)	≤ 1 a settimana = 1 punto
**11.** Quante porzioni di carne processata (hamburger, salsicce, würstel o salumi) consuma a settimana? (porzione = 100–150 g)	≤ 1 a settimana = 1 punto
**12.** Quante porzioni di carne di pollo, tacchino, coniglio o carne magra di maiale (lombo, filetto) consuma a settimana? (1 pezzo o porzione = 100–150 g)	≥ 3 alla settimana = 1 punto
**13.** Quante bevande gassate e/o zuccherate (bibite, cola, tonica, bitter) consuma a settimana?	< 1 a settimana = 1 punto
**14.** Quante porzioni di frutta secca a guscio (noci, nocciole, mandorle, arachidi o pistacchi) consuma a settimana? (porzione = 30 g)	≥ 3 a settimana = 1 punto
**15.** Quante volte consuma dolci come biscotti, dolci al cucchiaio, pasticcini o torte a settimana?	< 2 a settimana = 1 punto
**16.** Quanti latticini (latte, yogurt, formaggi) consuma al giorno?	≥ 3 al giorno = 1 punto
**17.** Quante porzioni di burro, margarina o panna consuma a settimana? (porzione = 12 g)	< 1 a settimana = 1punto

### Questionnaire validity and reliability

A total of 116 Italian pregnant women participated in the validation study. The socio-demographic characteristics of the sample are reported in Table 2. The mean age of the participants was 33.5 ± 4.4 years. The majority were born in Northen Italy (72.4%), while the remaining 27.6% were born in Central or Southern Italy or in the Islands. Most women held a bachelor’s or a master’s degree (61%), were full-time employed (46%) or on maternity leave (41%), and 20% declared having a background in nutrition. Regarding pregnancy-related characteristics, the majority of participants were in the third trimester of pregnancy (56%) and nulliparous (68%). In the overall sample, medium adherence to the MD was observed in 59% of participants, with a mean score of 8.61 ± 1.30, 38% of the sample showed low adherence to the MD, with a mean score of 4.55 ± 1.34, while only 3% of the enrolled pregnant women had high adherence, with a mean score of 12.67 ± 0.58.

**Table 2:** Participants’ sociodemographic characteristics (*n* = 116)

Participants’ characteristics (*n* = 116)	
Age (years)	33.5 ± 4.4
*Gestation at questionnaire administration*	
First trimester (< 13 + 6 weeks)	6 (5.2%)
Second trimester (14 + 0–27 + 6 weeks)	45 (38.8%)
Third trimester (≥ 28 weeks)	65 (56.0%)
*Nulliparity*	
Nulliparous	79 (68.1%)
Multiparous	37 (31.9%)
*Italian geographical area of birth*	
Northwest (Aosta Valley, Piedmont, Liguria, Lombardy)	26 (22.4%)
Northeast (Emilia–Romagna, Veneto, Trentino–South Tyrol, Friulia Venezia Giulia)	58 (50.0%)
Central (Tuscany, Marche, Umbria, Lazio)	5 (4.3%)
South (Abruzzo, Molise, Campani, Puglia, Basilicata, Calabria)	19 (16.4%)
Islands (Sicily and Sardinia)	8 (6.9%)
*Educational level*	
Pre-graduated	21 (10.4%)
Graduated	71 (61.2%)
Post-graduated	24 (20.7%)
*Employment*	
Unemployed	5 (4.3%)
Full-time employee	53 (45.7%)
Part-time employee	10 (8.6%)
Employed currently on maternity leave	48 (41.4%)
*Nutritional background*	
Yes	23 (19.8%)
No	93 (80.2%)
*Adherence to the MD*	
Low (≤ 6 pt.)	44 (37.9%)
Medium (7-11pt.)	69 (59.5%)
High (≥ 12 pt.)	3 (2.6%)

Data are expressed as mean ± SD or as an absolute number (frequency)

The total sample obtained a mean score of 7.17 ± 2.52 at the first administration (t1) of the ITpreg-MEDAS questionnaire (Table 3), indicating an overall medium level of adherence to the MD. A slightly higher mean score (7.43 ± 2.57) was observed at the first administration in participants who declared having a nutritional background in comparison to the mean score (7.11 ± 2.52) of participants without a background in nutrition. However, after controlling for sociodemographic variables, no statistically significant difference was observed between the two groups (*p* = 0.433) (Table 3).

A total of 111 pregnant women completed the second administration (t2) of the questionnaire. No meaningful differences in baseline characteristics were observed between participants who completed both questionnaire administrations and those lost to follow-up. Their mean adherence scores were similar between baseline and the second administration. Based on these findings the questionnaire showed good temporal stability (Pearson’s *r* = 0.756, 95% CI: 0.664–0.826; intraclass correlation coefficient, ICC = 0.671, 95% CI: 0.644–0.695), indicating strong stability over time. Internal consistency was acceptable, with a Cronbach’s alpha coefficient of 0.623 (Table 3).

**Table 3:** Construct validity, internal consistency, and test-retest reliability

Score range	Baseline score (t1) (*n* = 116)	Nutritional background (*n* = 23)	No nutritional background (*n* = 93)	*p*-value*	Baseline score (t1) (*n* = 111)	Follow-up score (t2) (*n* = 111)	Correlation	ICC	Cronbach’s α
	Mean ± SD	Mean ± SD	Mean ± SD		Mean ± SD	Mean ± SD	*R* (95% CI)	ICC (95% CI)	
0–17	7.17 ± 2.52	7.43 ± 2.57	7.11 ± 2.52	0.433	7.27 ± 2.52	7.36 ± 2.66	0.756 (0.664–0.826)	0.671 (0.644–0.695)	0.623

SD, standard deviation; ICC, intraclass correlation coefficient; CI, confidence interval; * The *p*-value refers to the comparison between participants with a nutritional background and with no nutritional background and was calculated using Welch’s independent-samples t-test

## Discussion

The findings support that the Italian version of the pregnancy-adapted Mediterranean Diet Screener (ITpreg-MEDAS) is a valid and culturally appropriate tool for assessing adherence to the MD during pregnancy. Although several questionnaires designed to assess adherence to the MD have been validated in the Italian population [32–35], to the best of our knowledge, this is the first time that a tool is validated for the pregnant population.

Given the well-established beneficial effects of the MD on maternal and pregnancy-related outcomes [5, 12], the availability of valid and reliable tools specifically tailored to pregnant women’s nutritional requirements is crucial for both clinical practice and research. In this context, the process of translation and cross-cultural adaptation allows the validation of the same tools across different countries while preserving their psychometric properties and ensuring equivalence between versions. This approach promotes a better knowledge exchange on a global level, supporting homogeneity and standardisation of data and increasing the comparability among studies conducted in different settings [26, 36]. Moreover, in the case of questionnaires focused on dietary habits, cross-cultural adaptation facilitates the development of instruments that are aligned with national dietary guidelines and the country’s culinary traditions, thereby improving the accuracy of dietary assessment.

Throughout the process of translation and cross-cultural adaptation, particular attention was paid to align food-related terminology and examples with common Italian culinary tradition and practices, as well as with the Italian FBDGs [31]. During the pre-testing phase, participants reported high clearness and comprehensibility of the ITpreg-MEDAS. One of the main difficulties identified concerned the alignment of habitual eating habits with the reference portion size specified in questionnaire. This finding reflects a well-recognised limitation of dietary assessment tools related to portion size estimation, particularly among individuals who are not accustomed to quantifying food consumption [37, 38]. In addition, some participants suggested the inclusion of visual aids for food examples and portion sizes. This may represent a valuable direction for the future implementation of the instrument, in particular in the context of digital applications. In fact, this implementation has been employed in similar questionnaires for the general population, including the MEDI-Lite questionnaire [39, 40]. Overall, these findings support the cultural adequacy and practical feasibility of the ITpreg-MEDAS in the Italian pregnant population.

Our findings are consistent with previous validation studies of short dietary assessment questionnaires. Regarding psychometric properties, the questionnaire showed adequate internal consistency and temporal stability. Although values above 0.70 are commonly considered adequate [29, 41], Cronbach’s alpha values greater than 0.60 have been deemed acceptable in previous validation studies [30, 42, 43]. Furthermore, the internal consistency measured in this study is aligned with results reported for the translation, cross-cultural adaptation, and validation of other short questionnaires designed to assess dietary habits [36, 44]. The temporal stability of the questionnaire was also considered satisfactory, in line with the results of test-retest analysis which suggested that the instrument provides stable measurements over time. Construct validity was explored by comparing scores of the questionnaire between pregnant women with and without nutritional background. As expected, participants with a nutritional background achieved a slightly higher mean score. In contrast to previous studies on nutrition knowledge [45, 46], no statistically significant differences were observed between groups, even after adjustment for relevant sociodemographic factors. This finding may reflect the limited number of participants with a nutritional background, as well as the influence of other unmeasured determinants of dietary behaviors during pregnancy, since nutritional knowledge does not necessarily translate into healthier food choices.

The data collected showed that the majority of participants (97%) reported a low or medium adherence to the MD during pregnancy. Although the mean score falls within the range defined by the questionnaire as “medium adherence” (7–11 points), this finding is concerning, as it is closer to the low-adherence category than to high adherence. These findings suggest that Italian pregnant women included in the study may have poor dietary habits. Even if these results cannot be considered representative of the whole Italian pregnant population, comparable findings have been reported in the literature. Similar patterns of suboptimal adherence to the MD during pregnancy have been reported in previous studies [47, 48], also at Italian level [18, 49, 50]. However, direct comparisons should be interpreted with caution, as previous studies used different adherence indices, scoring systems, and cut-off values, which were not specifically validated for the Italian pregnant population. These findings highlight a consistent gap between nutritional recommendations and actual dietary practices among pregnant women. Considering this context, there is an even greater need for harmonised, pregnancy-adapted dietary assessment tools that can be used in targeted interventions to enhance dietary practices on a global scale.

This study has both strengths and limitations. Among the limitations, the generalisability of the questionnaire is limited by the characteristics of the sample, which was predominantly of medium-high socio-demographic status, as most participants held a university degree and were employed full-time. Moreover, most women resided in Northern Italy. In addition, although all participants were of Italian nationality, ethnicity and cultural background was not collected. Since dietary habits and adherence to the MD may vary across different ethnic and cultural backgrounds, the lack of this information may limit the interpretation and generalizability of our findings. Taken together, these aspects highlight the need for future studies involving a more socio-economically and geographically diverse Italian population. Dietary information was also self-reported and may therefore be subject to recall bias. Another limitation concerns the enrolment setting: although the recruitment targeted both community and private settings, most of the women enrolled attended public health facilities.

Regarding the psychometric properties of the tool, the absence of significant differences in questionnaire scores according to nutritional background, even after adjustment for sociodemographic factors, should be interpreted with caution. This finding may partly reflect the small number of participants with formal nutrition training and the influence of other unmeasured determinants of dietary behaviour during pregnancy. Future studies should therefore include larger and more balanced subgroups and investigate additional factors associated with adherence to the MD. Although the original Spanish questionnaire had already been validated against an independent dietary assessment instrument in a comparable population of pregnant women, future studies should assess criterion validity specifically in the Italian context, as measurement properties may vary across cultural and dietary settings.

Despite these limitations, the study has several strengths. The ITpreg-MEDAS is the first pregnancy-adapted tool for the assessment of adherence to the MD validated in a sample of Italian pregnant women. The translation, cross-cultural adaptation, and validation analysis were conducted in accordance with guidelines and supervised by a multidisciplinary team of experts, enhancing the scientific rigor and reliability of the process.

From a practical perspective, considering the clinically associated benefits of MD, the ITpreg-MEDAS may represent a useful screening tool in both clinical practice and epidemiological studies to rapidly assess adherence to the MD during pregnancy and to identify women who may benefit from targeted nutritional interventions.

## Conclusions

Dietary patterns during pregnancy play a pivotal role in maternal health and foetal development, influencing both short- and long-term outcomes. In this context, the MD represents a dietary model with well-recognized beneficial effects on pregnancy outcomes. The ITpreg-MEDAS questionnaire, derived from the original Spanish preg-MEDAS and subsequently translated, cross-culturally adapted, and validated for the Italian context, is a reliable and valid tool for assessing adherence to the MD among Italian pregnant women. This instrument may support both research and public health strategies aimed at improving maternal nutrition. Future studies should evaluate the predictive validity of the ITpreg-MEDAS in relation to maternal and offspring health outcomes.

## Supplementary Information

Below is the link to the electronic supplementary material.

Supplementary Material 1

## Data Availability

The datasets used and/or analysed during the present study are available from the corresponding author on reasonable request.
