# Supplementary material for: Validation and cultural adaptation of the pregnancy-adapted Mediterranean Diet Adherence Screener (ITpreg-MEDAS) in Italian pregnant women
Source: Eur J Nutr. 2026 Aug 29;65(6):247. doi: 10.1007/s00394-026-04087-4 (PMC13526079; doi:10.1007/s00394-026-04087-4)
Supplement: Supplementary file 1 [file 394_2026_4087_MOESM1_ESM.docx]

**Supplementary Table 1.** Process of translation into Italian and cultural adaption of the questionnaire pregnancy-adapted Mediterranean Diet Adherence Screener (preg-MEDAS).

|  | **Original Version  (preg-MEDAS)** | **T1 and T2** | **T12** | **BT1 and BT2** | **P-FTL** |
| --- | --- | --- | --- | --- | --- |
| **1** | ¿Cuántas cucharas de aceite de oliva consume en total al día? (incluyendo el usado para freír, comidas fuera de casa, ensaladas, etc.): | **T1:** Quanti cucchiai di olio di oliva consuma in totale al giorno? (includendo quello usato per friggere, le pietanze consumate fuori casa, le insalate, etc.) **T2:** Quanti cucchiai di olio di oliva consuma in totale al giorno? (Includendo quello usato per friggere, pasti fuori casa, insalate, etc.) | Quanti cucchiai di olio di oliva consuma in totale al giorno? (includendo quello usato per friggere, i pasti consumati fuori casa, le insalate, etc.) | **BT1:** ¿Cuántas cucharas de aceite de oliva consume en total al día? (incluyendo el que usa para freír, las comidas consumidas fuera de casa, las ensaladas, etc.)  **BT2:** ¿Cuántas cucharadas de aceite de oliva consume en total al día? (Incluyendo el usado para freír, en las comidas fuera de casa, las ensaladas, etc.) | Quanti cucchiai di olio d’oliva consuma in totale al giorno? (includendo quello usato per cucinare, friggere, i pasti consumati fuori casa, le insalate, etc.) |
| **2** | ¿Cuántas raciones de verdura u hortalizas consume al día? (las guarniciones o acompañamientos= 1/2 ración) 1 ración = 200 g: | **T1:** Quante porzioni di verdura e ortaggi consuma al giorno? (guarnizioni o contorni = ½ porzione; 1 porzione = 200 g)  **T2:** Quante razioni di verdura o ortaggi consuma al giorno (i contorni=1/2 razione) 1 razione = 200 g | Quante porzioni di verdura e ortaggi consuma al giorno? 1 porzione = 200 g (contorni = ½ porzione) | **BT1:** ¿Cuántas raciones de verduras y hortalizas consume diariamente?  1 ración = 200 g (acompañamiento = ½ ración)  **BT2:** ¿Cuántas porciones de verdura y hortalizas consume al día? 1 porción = 200 g ( guarnición= ½ porción) | Quante porzioni di verdura e ortaggi consuma al giorno? 1 porzione = 200 g (contorni = ½ porzione) |
| **3** | ¿Cuántas piezas de fruta consume al día?: | **T1:** Quanti frutti consuma al giorno?  **T2:** Quanti frutti consuma al giorno? | Quanti frutti consuma al giorno? | **BT1:** *¿Qué cantidad de fruta consume diariamente?*  **BT2:** ¿Cuántas piezas de fruta consume al día? | Quanti frutti consuma al giorno? |
| **4** | ¿Cuántas veces a la semana consume los vegetales cocinados, la pasta, arroz u otros platos aderezados con salsa de tomate, ajo, cebolla o puerro elaborada a fuego lento con aceite de oliva (sofrito) | **T1:** Quante volte a settimana consuma verdure cotte, pasta, riso o altri piatti conditi con salsa di pomodoro, aglio, cipolla o porro cotta a fuoco basso con olio di oliva (soffritto)? **T2:** Quante volte a settimana consuma verdure cotte, pasta, riso o altri piatti conditi con salsa di pomodoro, aglio, cipolla o porro cucinata a fuoco lento con olio d'oliva (soffritto)? | Quante volte a settimana consuma verdure cotte, pasta, riso o altri piatti conditi con salsa di pomodoro, aglio, cipolla o porro cotta a fuoco basso con olio di oliva (soffritto)? | **BT1:** *¿Cuántas veces por semana consume verduras cocidas, pasta, arroz u otros platos aliñados con salsa de tomate, ajo, cebolla o puerro, cocidos a fuego lento con aceite de oliva (sofrito)?*  **BT2:** ¿Cuántas veces a la semana consume verduras cocidas, pasta, arroz u otros platos condimentados con salsa de tomate, ajo, cebolla o puerro cocinada a fuego lento con aceite de oliva (sofrito)? | Quante volte a settimana consuma vegetali cotti, pasta, riso o altri piatti conditi con salsa di pomodoro, aglio, cipolla o porro cotta a fuoco basso con olio di oliva (soffritto)? |
| **5** | ¿Cuántas raciones de cereales y alimentos integrales (pan, arroz, pasta) consume a la semana?: | **T1:** Quante porzioni di cereali e alimenti integrali (pane, riso, pasta) consuma a settimana? **T2:** Quante razioni di cereali e alimenti integrali (pane, riso, pasta) consuma a settimana? | Quante porzioni di cereali e alimenti integrali (pane, riso, pasta) consuma a settimana? | **BT1:** *¿Cuántas raciones de cereales y alimentos integrales (pan, arroz y pasta) consume semanalmente?*  **BT2:** ¿Cuántas porciones de cereales y alimentos integrales (pan, arroz, pasta) consume a la semana? | Quante porzioni di cereali e alimenti integrali (pane, riso, pasta) consuma a settimana? |
| **6** | ¿Cuántas raciones de pan, arroz y/o pasta refinados consume a la semana?: | **T1:** Quante porzioni di pane, riso e/o pasta raffinati consuma a settimana?  **T2:** Quante razioni di pane, riso e/o pasta raffinate consuma a settimana? | Quante porzioni di pane, riso e/o pasta raffinati consuma a settimana? | **BT1:** *¿Cuántas raciones de pan, arroz y/o pasta refinados consume semanalmente?*  **BT2:** ¿Cuántas porciones de pan, arroz, y/o pasta refinados consume a la semana? | Quante porzioni di pane, riso e/o pasta raffinati (non integrali) consuma a settimana? |
| **7** | ¿Cuántas raciones de legumbres consume a la semana? (1 plato o ración de 150 g.) | **T1:** Quante porzioni di legumi consuma a settimana? (1 piatto o una porzione da 150 g) **T2:** Quante razioni di legumi consuma a settimana? (1 piatto o razione da 150 g) | Quante porzioni di legumi consuma a settimana? (1 piatto o porzione da 150 g). | **BT1:** *¿Cuántas raciones de legumbres consume semanalmente? (1 plato o una ración de 150 g)*  **BT2:** ¿Cuántas porciones de legumbres consume a la semana? ( 1 plato o porción de 150 g) | Quante porzioni di legumi consuma a settimana? (1 piatto o porzione = 150 g) |
| **8** | ¿Cuántas raciones de pescado - mariscos consume a la semana? (1 plato, pieza o ración= 100 - 150 g. de pescado o 4 - 5 piezas o 200 g. de marisco): | **T1:** Quante porzioni di pesce o frutti di mare consuma a settimana? (1 piatto, un pezzo o una porzione = 100 – 150 g di pesce o 4-5 pezzi o 200 g di frutti di mare) **T2:** Quante razioni di pesce - frutti di mare consuma a settimana? (1 piatto, pezzo o razione = 100 - 150 g. di pesce o 4 - 5 pezzi o 200 g. di frutti di mare) | Quante porzioni di pesce o frutti di mare (molluschi e crostacei) consuma a settimana? (1 piatto, pezzo o porzione = 100 – 150 g di pesce; 4-5 pezzi o 200 g di frutti di mare) | **BT1:**  *¿Cuántas raciones de pescado o marisco (moluscos y crustáceos) consume semanalmente? (1 plato, trozo o ración = 100-150 g de pescado, 4-5 trozos o 200 g de marisco)*  **BT2:** ¿Cuántas porciones de pescado o mariscos consume a la semana? ( 1 plato, pedazo o porción = 100-150 g de pescado; 4-5 pedazos o 200 g de marisco) | Quante porzioni di pesce o frutti di mare (molluschi e crostacei) consuma a settimana? (1 piatto, pezzo o porzione = 100 – 150 g di pesce; 4-5 pezzi o 200 g di frutti di mare) |
| **9** | ¿Come una ración (100-150 g) de pescado azul pequeño a la semana | **T1:** Consuma una porzione (100 – 150g) di pesce azzurro di piccola taglia a settimana? **T2:** Mangia una razione (100-150 g) di pesce azzurro piccolo a settimana? | Consuma una porzione (100 – 150g) di pesce azzurro di piccola taglia (es. sardine, sgombro) a settimana? | **BT1:** *¿Consume una ración (100-150 g) de pescado azul de pequeñas dimensiones (p.ej., sardinas, caballa, etc.) por semana?*  **BT2:** ¿Consume una porción (100-150 g) de pescado azul de pequeño tamaño (ej. Sardinas, caballa) a la semana? | Consuma una porzione (100 – 150 g) di pesce azzurro di piccola taglia (es. sardine, sgombro) a settimana? |
| **10** | ¿Cuántas raciones de carne roja: ternera, buey, caballo, potro, cordero, partes no magras del cerdo o pato) consume a la semana? (ración= 100 - 150 g.) | **T1:** Quante porzioni di carne rossa (vitello, manzo, cavallo, puledro, agnello, parti non magre del maiale o anatra) consuma a settimana (porzione = 100 – 150 g) **T2:** Quante razioni di carne rossa: vitello, bue, cavallo, puledro, agnello, parti non magre del maiale o anatra consuma a settimana? (Razione = 100 - 150 g.) | Quante porzioni di carne rossa (vitello, manzo, cavallo, puledro, agnello, parti non magre del maiale o anatra) consuma a settimana (porzione = 100 – 150 g) | **BT1:** *¿Cuántas raciones de carne roja (ternera, vaca, caballo, potro, cordero, partes con grasa de cerdo o pato) consume semanalmente? (ración = 100-150 g)*  **BT2:** ¿Cuántas porciones de carne roja (ternera, buey, caballo, potro, cordero, partes no magras del cerdo o pato) consume a la semana? (Porción = 100-150 g) | Quante porzioni di carne rossa (vitello, manzo, cavallo, puledro, agnello, parti non magre del maiale o anatra) consuma a settimana (porzione = 100 – 150 g) |
| **11** | ¿Cuántas raciones de carne procesada: hamburguesas, salchichas o embutidos consume a la semana? (ración= 100 - 150 g.): | **T1:** Quante porzioni di carne processata (hamburger, salsicce o salumi) consuma a settimana? (porzione = 100 – 150 g) **T2:** Quante razioni di carne processata: hamburger, salsicce o insaccati consuma a settimana? (razione = 100 - 150 g.) | Quante porzioni di carne processata (hamburger, salsicce o salumi) consuma a settimana? (porzione = 100 – 150 g) | **BT1:** *¿Cuántas raciones de carne procesada (hamburguesa, salchichas o embutidos) consume semanalmente? (ración = 100-150 g)*  **BT2:** ¿Cuántas porciones de carne procesada (hamburguesas, salchichas o chacinas) consume a la semana? ( Porción = 100-150 g) | Quante porzioni di carne processata (hamburger, salsicce, würstel o salumi) consuma a settimana? (porzione = 100 – 150 g) |
| **12** | ¿Cuántas raciones de carne de pollo, pavo, conejo o carne magra (lomo, solomillo) consume a la semana? (carne de pollo= 1 pieza o ración de 100 - 150 g.) | **T1:** Quante porzioni di carne di pollo, tacchino, coniglio o carne magra di maiale (lombo, filetto) consuma a settimana? (1 pezzo o porzione di 100 -150 g) **T2:** Quante razioni di carne di pollo, tacchino, coniglio o carne magra (lombata/lombo/lonza, filetto) consuma a settimana? (carne di pollo = 1 pezzo o razioni da 100 - 150 g.) | Quante porzioni di carne di pollo, tacchino, coniglio o carne magra di maiale (lombo, filetto) consuma a settimana? (1 pezzo o porzione di 100 -150 g) | **BT1:** *¿Cuántas raciones de carne de pollo, pavo, conejo o carne con poca grasa de cerdo (lomo o filete) consume semanalmente? (1 ración o trozo = 100-150 g)*  **BT2:** ¿Cuántas porciones de carne de pollo, pavo, conejo o carne magra de cerdo (lomo, solomillo) consume a la semana? ( 1 pedazo o porción de 100-150 g) | Quante porzioni di carne di pollo, tacchino, coniglio o carne magra di maiale (lombo, filetto) consuma a settimana? (1 pezzo o porzione = 100 -150 g) |
| **13** | ¿Cuántas bebidas carbonatadas y/o azucaradas (refrescos, colas, tónicas, bitter) consume a la semana | **T1:** Quante bibite gassate e/o zuccherate (bevande analcoliche, cola, tonica, bitter) consuma a settimana? **T2:** Quante bevande gassate e/o zuccherate (bibite, cola, tonica, bitter) consuma a settimana? | Quante bevande gassate e/o zuccherate (bibite, cola, tonica, bitter) consuma a settimana? | **BT1:** *¿Cuántas bebidas con gas y/o azucaradas (refrescos, cola, tónica, bitter, etc.) consume semanalmente?*  **BT2:** ¿Cuántas bebidas con gas y/o azucaradas (refrescos, cola, tónica, bitter) consume a la semana? | Quante bevande gassate e/o zuccherate (bibite, cola, tonica, bitter) consuma a settimana? |
| **14** | ¿Cuántas veces consume frutos secos (nueces, avellanas, almendras, cacahuetes o pistachos) a la semana? (ración 30 g.) | **T1:** Quante volte consuma frutta secca a guscio (noci, nocciole, mandorle, arachidi o pistacchi) a settimana? (porzione 30 g) **T2:** Quante volte a settimana consuma frutta secca (noci, nocciole, mandorle, arachidi o pistacchi) (razione 30 g.) | Quante volte consuma frutta secca a guscio (noci, nocciole, mandorle, arachidi o pistacchi) a settimana? (porzione 30 g) | **BT1:** *¿Cuántas veces consume frutos secos (nueces, avellanas, almendras, cacahuetes o pistachos) por semana? (ración = 30 g)*  **BT2:** ¿ Cuántas veces consume frutos secos con cáscara ( nueces, cacahuetes, almendras o pistachos) a la semana? (Porción 30 g) | Quante porzioni di frutta secca a guscio (noci, nocciole, mandorle, arachidi o pistacchi) consuma a settimana? (porzione = 30 g) |
| **15** | ¿Cuántas veces consume repostería como galletas, flanes, dulces o pasteles a la semana?: | **T1:** Quante volte consuma dolci come biscotti, budini, pasticcini o torte a settimana? **T2:** Quante volte consuma dolci come biscotti, budini, dolciumi o torte a settimana? | Quante volte consuma dolci come biscotti, budini, pasticcini o torte a settimana? | **BT1:** *¿Cuántas veces consume postres como galletas, flanes, dulces o tartas por semana?*  **BT2:** ¿Cuántas veces consume a la semana dulces como galletas, flanes, pasteles o tartas? | Quante volte consuma dolci come biscotti, dolci al cucchiaio, pasticcini o torte a settimana? |
| **16** | ¿Cuántos lácteos (leche, yogurt, quesos) toma al día? | **T1:** Quanti latticini (latte, yogurt, formaggi) consuma al giorno? **T2:** Quanto latte e latticini (latte, yogurt, formaggi) consuma al giorno? | Quanti latticini (latte, yogurt, formaggi) consuma al giorno? | **BT1:** *¿Cuántas lácteos (leche, yogurt, quesos, etc.) consume al día?*  **BT2:** *¿Cuántos lácteos ( leche, yogures, quesos) consume al día?* | Quanti latticini (latte, yogurt, formaggi) consuma al giorno? |
| **17** | ¿Cuántas raciones de mantequilla, margarina o nata consume a la semana? (porción individual = 12 g.): | **T1:** Quante porzioni di burro, margarina o panna consuma a settimana? (porzione singola = 12 g) **T2:** Quante razioni di burro, margarina o panna consuma a settimana (razione individuale = 12 g.): | Quante porzioni di burro, margarina o panna consuma a settimana? (porzione = 12 g) | **BT1:** *¿Cuántas raciones de mantequilla, margarina o nata consume semanalmente? (ración = 12 g)*  **BT2:** *¿Cuántas porciones de mantequilla, margarina o nata consume a la semana? ( Porción = 12 g)* | Quante porzioni di burro, margarina o panna consuma a settimana? (porzione = 12 g) |
